# Sugar Antennae for Guidance Signals: Syndecans and Glypicans Integrate Directional Cues for Navigating Neurons

**DOI:** 10.1100/tsw.2006.202

**Published:** 2006-08-25

**Authors:** Christa Rhiner, Michael O. Hengartner

**Affiliations:** ^1^ Neuroscience Center Zurich, University of Zurich, CH-8057, Switzerland; ^2^ Institute of Molecular Biology, University of Zurich, CH-8057, Switzerland

**Keywords:** axon guidance, neuronal migration, heparan sulfate proteoglycans (HSPGs), syndecan, C. *elegans*, glypican

## Abstract

Attractive and repulsive signals guide migrating nerve cells in all directions when the nervous system starts to form. The neurons extend thin processes, axons, that connect over wide distances with other brain cells to form a complicated neuronal network. One of the most fascinating questions in neuroscience is how the correct wiring of billions of nerve cells in our brain is controlled. Several protein families are known to serve as guidance cues for navigating neurons and axons. Nevertheless, the combinatorial potential of these proteins seems to be insufficient to sculpt the entire neuronal network and the appropriate formation of connections. Recently, heparan sulfate proteoglycans (HSPGs), which are present on the cell surface of neurons and in the extracellular matrix through which neurons and axons migrate, have been found to play a role in regulating cell migration and axon guidance. Intriguingly, the large number of distinct modifications that can be put onto the sugar side chains of these PGs would in principle allow for an enormous diversity of HSPGs, which could help in regulating the vast number of guidance choices taken by individual neurons. In this review, we will focus on the role of the cell surface HSPGs syndecan and glypican and specific HS modifications in promoting neuronal migration, axon guidance, and synapse formation.

Nerve cells communicate over an immense number of established contacts called synapses. The formation of such connections in neural maps is complex and occurs over several intermediate steps. In an early phase, neurons that are generated from neuronal stem cells have to migrate from their place of birth to the place at which they are destined to differentiate and function. For example, large populations of neurons in the developing mammalian brain are known to travel extensively along different routes in parallel to the brain surface to reach their target area. Other neurons, emigrate radially along specialized glia cells and give rise to the multiple layers of the human cortex[1]. In the latter case, the processes of radial glia are used as guidewires for the migrating neurons. This form of migration, however, is a modern invention, unique to higher vertebrates. In classical forms of neuronal migration, the neurons are guided by secreted or cell surface–bound attractive or repulsive cues and by interactions with extracellular matrix proteins. In this way, a migrating neuron is exposed to a wealth of different stimuli and molecular gradients while navigating to its often-distant target position. How does a neuron integrate all these signals? How is it able to veer slightly more to the right than its neighbor who is heading the same general direction? Why does it stop after its remarkable journey at precisely its intended final location? These questions reappear later, when neurons start to send out axons that have to establish synaptic contacts with a target cell over a long distance. While migrating neurons and axonal growth cones do not possess a brain GPS (global positioning system) device, recent findings suggest that they utilize at least one sort of signal-integrating and fine-tuning instrument: heparan sulfate proteoglycans (HSPGs) that are mounted antennae-like on the cell surface and regulate guidance signals via their sugar side chains. HSPGs are a family of highly conserved PGs that consist of membrane-anchored or secreted protein cores to which several highly modified HS polysaccharide chains are attached[2,3,4]. Syndecans and glypicans are the two main cell surface HSPGs, whereas agrin and perlecan are found in the extracellular matrix (Fig. 1). Here, we will focus mainly on the role of syndecans and glypicans in nervous system development.

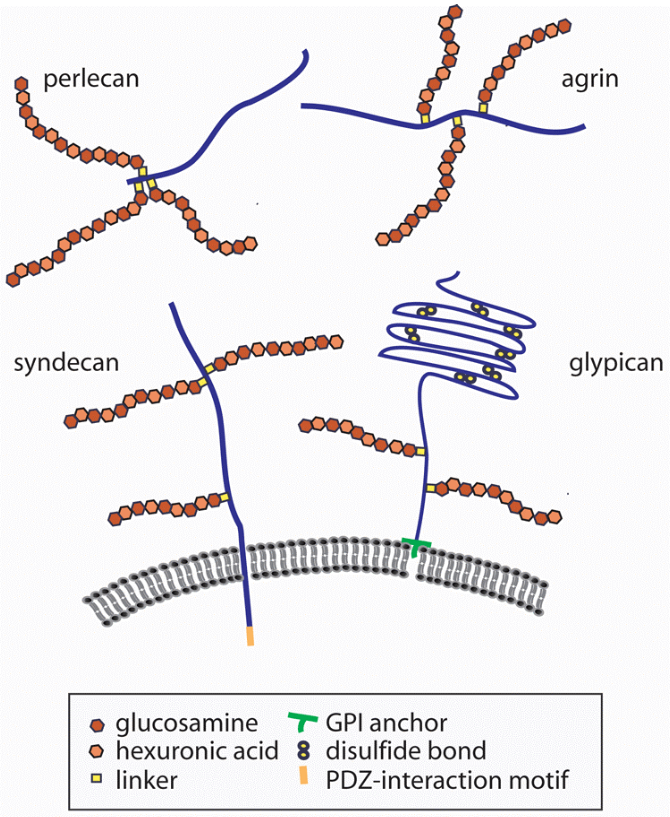

**FIGURE 1.** Cell surface and extracellular HSPGs. Syndecans and glypicans are integral membrane proteins. Syndecan is a single-pass transmembrane protein with a short conserved cytoplasmic domain containing a C-terminal PDZ-interacting motif. HS side chains are attached to the extracellular domain of the core protein at conserved Serine residues. Yellow boxes represent a typical tetrasaccharide linker region, which connects the HS chain to the serine. The glypican core protein forms a globular domain, which is stabilized by conserved disulfide bonds and linked to the cell surface via a GPI anchor. In contrast to syndecan, the major HS attachment sites in glypican are located proximal to the membrane. Perlecan and agrin are large multidomain proteins that contain several HS attachment sites. They both exist as multiple isoforms that are generated by alternative splicing and are found in basement membranes of many tissues.

## SIGNALS FROM OUTER SPACE: EXTRACELLULAR BINDING PARTNERS OF HSPGs

The highly modified HS chains of HSPGs are known to interact with an enormous range of extracellular ligands, and therefore control multiple cellular responses in both developmental and pathological processes[5,6]. Growth factors, morphogens, axon guidance cues, antithrombin, and extracellular matrix components, all seem to end up in the tentacles of HS side chains.

Fibroblast growth factors (FGFs) are probably the best-studied molecules that directly bind to HS and where the specific HS sequence required for ligand binding is known[7]. Crystal structures have shown that HS, FGF, and its receptor form a ternary complex on the cell surface[8,9]. Since HSPGs are essential for efficient FGF signaling, as well as for other ligand-receptor interactions, they are thought to serve as coreceptors in several signaling pathways[2]. HSPGs are also known to interact with axon guidance cues. The presence of HS increases the affinity of Slit, a repellent secreted by midline cells, for its receptor roundabout (Robo), which is compatible with a coreceptor function of HSPGs. If cultured olfactory bulb axons are treated with heparinase to remove HS from the cell surface, they no longer respond to the repulsive effect of Slit[10]. Moreover, biochemical copurification has revealed a direct binding of Slit to glypican-1[11,12] and of Slit and Robo to *Drosophila* syndecan (Sdc)[13]. The axon guidance cue semaphorin 5A was also found to interact physically with syndecan-3 in the rat brain[14] as well as anosmins, which are conserved secreted proteins that regulate cell migration and axon branching. A recent study has shown that the *C. elegans* anosmin-1 KAL-1 binds specifically to modified HS of syndecan and glypican[15].

Ephrins and Ephrin receptors represent yet another axon guidance system that seems to cooperate with HSPGs. EphB2 can tyrosine-phosphorylate the cytoplasmic domain of syndecan-2, a signaling event involved in the maturation of dendritic spines[16]. This study points to the role of certain HSPGs as possible independent signal transducers. On the other hand, syndecans on muscle cells are also thought to act in *trans* as ligands in the case of their interaction with the neuronal receptor protein tyrosine phosphatase (RPTP) LAR, which binds to the HS chains of *Drosophila* syndecan and glypican with nanomolar affinity[17].

Morphogens are secreted signaling molecules that differentially regulate cell fate in a concentration-dependent manner during development. HS chains help generating morphogen gradients and are required for proper signaling levels of the Wnt/Wingless (Wg), transforming growth factor-β (TGF-β) and Hedgehog (Hh) pathways in the *Drosophila* wing[18,19,20]. For the morphogen decapentaplegic (Dpp), a TGF-β family member, it was shown that extracellular diffusion of Dpp relies on the presence of cell surface glypicans, since Dpp failed to move across double mutant clones for both *Drosophila* glypicans, whereas Dpp signal transduction in a cell was dependent on dynamin-mediated endocytosis[21]. In this case, the HSPG glypican can be viewed as an active transporter that promotes Dpp movement along cell surfaces, thereby restricting its extracellular diffusion.

Besides morphogens, guidance cues, and growth factors, the list of molecules that require HS for activity includes matrix proteins (e.g., laminin, collagens, tenascin), coagulation factors (antithrombin), lipolytic proteins (apolipoprotein E, low-density lipoprotein), and components of the inflammatory response (chemokines, selectins)[3]. These interactions have to be interpreted with caution, however, since many HS ligands were identified using heparin, an unusual HS produced by mast cells, which contains extended N-sulfation domains.

## TRANSDUCING THE RIGHT FREQUENCY: SYNTHESIS OF HS ANTENNAE

How can several developmental progams rely on the same type of amplifying molecule without creating strong interferences? A closer look at how these molecular glyco-antennae are assembled provides some explanations for their versatile abilities to fine tune numerous signaling pathways.

HS synthesis occurs in the Golgi, where HS polymerases generate a nonsulfated sugar backbone, which is attached to the HS core protein (Fig. 2). HS chains begin with a characteristic tetrasaccharide linker (-Xyl-Gal-Gal-GlcA-) that is first attached to Serines within conserved Ser-Gly sequences on the protein core[3]. The linker tetrasaccharide serves as a primer for HS polymerases that add alternating residues of glucuronic acid (GlcA) and N-acetyl glucosamine (GlcNAc), the sugar building blocks of HS chains. The different sugar residues are all transferred from sugar nucleotides by individual Golgi enzymes[3] (Fig. 2). These early steps of HS assembly, such as the generation of sugar nucleotides or the synthesis of the tetrasaccharide linker are common to the synthesis of other glycosaminoglycans like chondroitin sulfate (CS) and dermatan sulfate (DS). As a result, genetic removal of the enzymes involved in these early synthetic steps causes a broad range of defects. In *C. elegans*, mutations in such "early" enzymes cause maternal-effect lethality due to defects in embryonic development and aberrant vulva formation, which has led to their classification as *squashed vulva* (*sqv*) genes (Fig. 2)[22].

Likewise, the genetic removal of HS polymerases causes a complete lack of HS, which interferes with multiple signaling pathways leading to severe patterning defects and lethality[18,23,24,25]. Mammals have five genes encoding for HS polymerases (Ext1, Ext2, Extl1, Extl2, Extl3)[26], whereas flies have three (*tout-velu* [*ttv*], *brother of ttv*, *sister of ttv*)[18,19,20], and worms two (*rib-1*, *rib-2*)[24,27]. Mice lacking exostosin 1 (Ext1) function exclusively in neurons die perinatally with severe brain patterning defects, including absence of olfactory bulbs, cerebellum, and corpus callosum[23]. Nonconditional Ext1 knock-out mice already die during gastrulation[28]. In zebrafish, the Ext family members Ext2 (dackel; dac) and Extl3 (boxer; box) play a role in the sorting of retinal axons in the optic tract and in fin and branchial arch development. Maternal contribution of Ext2 and Extl3 are thought to prevent early developmental defects in these mutants[29]. In general, these results clearly underscore the importance of HS for early development. But to gain insight into the meaning of the many possible HS modifications and how their combination and arrangement might lead to the vast density of information possibly specified by these sugar hieroglyphs, more selective ablations need to be analyzed.

## DECIPHERING THE HS CODE: ANALYSIS OF HS MODIFICATIONS

Once the HS precursor chains are assembled, the sugars are modified by several waves of HS-modifying enzymes. After deacetylation and N-sulfation of extensive regions by N-deacetylases/N-sulfotransferases (NDSTs), selected glucuronic acid units of the HS backbone are converted to iduronic acid by a glucuronyl C5-epimerase. Finally, several highly selective sulfotransferases (HS 2O-, 6O-, and 3O-sulfotransferases) introduce different O-sulfation motifs along the extending HS side chains[3] (Fig. 2). The HS modifications are not distributed uniformly; rather, microdomains of extensively modified sugars are surrounded by unmodified stretches. The result is an enormous diversity in HS chain sequences[4]. Not only can an almost endless combination of motifs be created this way, but also the length of the individual sugar chains is variable (from 50 to 150 disaccharides). By analyzing mutants that lack specific HS modifications, it has been possible to decipher the role played by many of these modified HS sequences.

N-sulfation is unique to HS and does not occur in related CS, DS, or keratan sulfate. Four mammalian NDSTs have been identified, which are likely to have different substrate-binding preferences, but only one isoform is present in *Drosophila* (*sulfateless*) and *C. elegans* (*hst-1*). *Ndst1* knock-out mice die at birth due to lung failure or partly *in utero* with skull and forebrain malformations[30,31]. A detailed analysis of *Ndst1*$^{-/-}$ brains revealed lack of the hippocampal and anterior commissure reminescent to loss of EXT1 function in the central nervous system[32]. *Sulfateless* flies are defective in morphogen (wg, hh, dpp) and growth factor signaling, and show segment polarity defects[21,33,34]. The drastic effects of loss of NDST function, comparable to complete lack of HS, indicate that the replacement of N-acetyl by N-sulfate groups in the HS sugar backbone is probably required for multiple signaling pathways in early development. It is conceivable that N-sulfations do not confer selective binding properties for certain

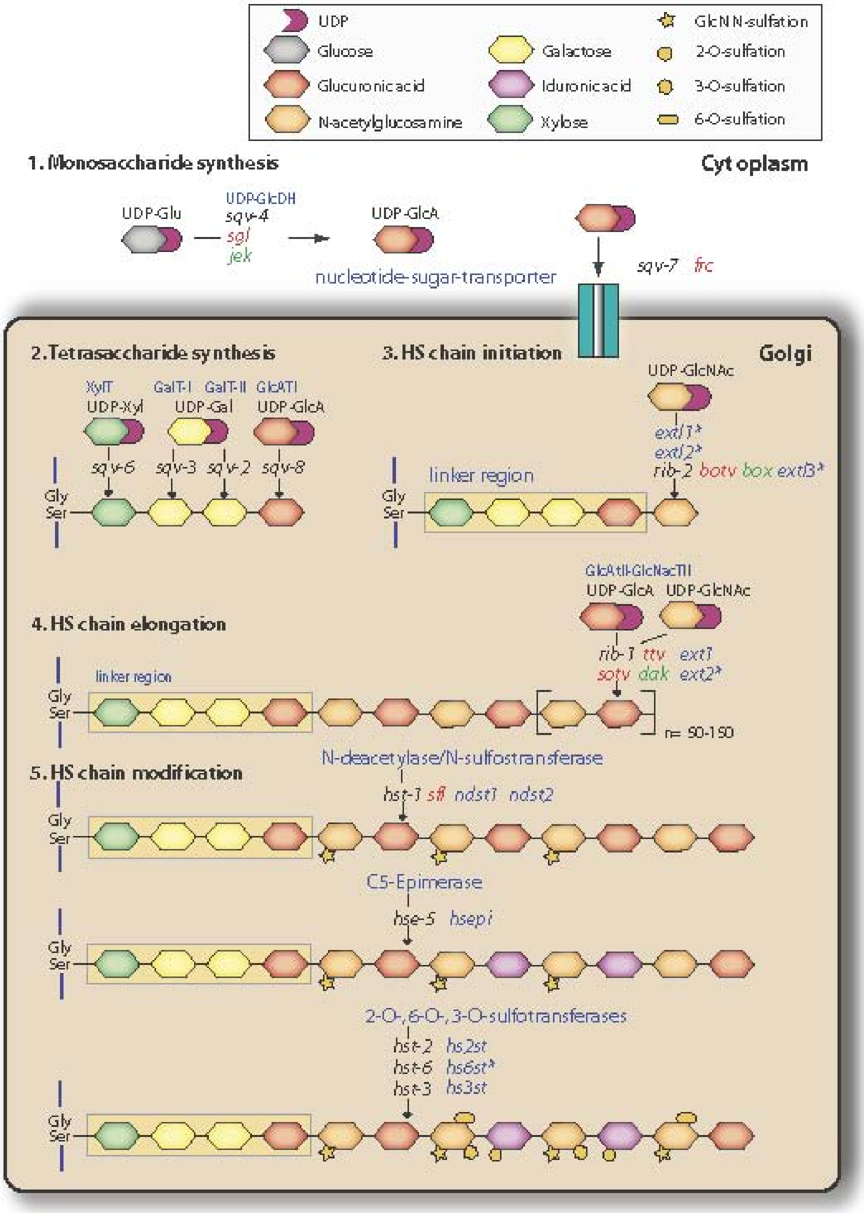

**FIGURE 2.** HS synthesis and modification. HS synthesis starts in the cytoplasm with the synthesis of monosaccharides, which are subsequently transported into the Golgi where they are incorporated into a conserved tetrassaccharide linker. HS polymerases catalyze the addition of glucuronic acid and N-acetyl glucosamine to the linker to form the HS sugar backbone. This backbone is later modified by a C5-epimerase and various sulfotransferases giving rise to complex modification patterns. Mutants for enzymes involved in HS synthesis and modification are shown in black (*C. elegans*), red (*Drosophila*), green (zebrafish), and blue (mouse). Enzymes are abbreviated as follows: GalTI, galactosyltransferase I; GalTII, galactosyltransferase II; GlcATI, glucuronyltransferase I; GlcATII, glucuronyltransferase II; GlcNACTI, N-acetylglucosamine transferase I; GlcNAcTII, N-acetylglucosamine transferase II; NDST, N-deacetylase/N-sulfotransferase; UDP-GlcDH, UDP-glucose dehydrogenase; XylT, xylosyltransferase. *botv*, *brother of tout velu*; *box*, *boxer*; *dak*, *dackel*; *ext, exostosin; extl, exostosin-like; frc, fringe connection*; *jek*, *jekyll*; *sfl*, *sulfateless*; *sgl*, *sugarless*; *sotv*, *sister of tout velu*; *sqv*, *squashed vulva*; *ttv*, *tout velu*. * No mouse knock-out available yet.

ligands, but rather serve as a structural hallmark required more generally for HS-ligand interactions. It is well documented that loss of NDST1 in mice does not only affect N-sulfation levels, but also strongly reduces the content of 2-O sulfated HS[35]. Therefore, the observed phenotype is not only caused by decreased N-sulfation, but results from a more broad alteration in the HS modification pattern.

Specific roles of HS motifs have emerged when research focused on enzymes involved in later events in HS modification. Biochemical analysis showed that different mammalian tissues indeed do have distinct HS fingerprints[35], but deciphering this potential "HS code" represents a major challenge. Lack of 2-O sulfation motifs in the mouse, for example, abolishes kidney formation, but also affects the development of the eyes and the skeleton[36]. RNAi of the 6-O sulfotransferase in flies interferes with tracheal development causing embryonic lethality[37]. In zebrafish, knock-down of HS6ST-2, but not HS6ST-1, impairs vascular branching[38]. Interestingly, vascular development and axon guidance are partially regulated by the same factors. 6-O sulfations could therefore be utilized by either system to control the formation of complex branching patterns.

In all organisms analyzed so far, there is only one gene encoding for the C5-epimerase. HS epimerase knock-out mice lack kidneys and show defects in lung and skeletal development leading to their death after birth[39]. In contrast, epimerase mutants in *C. elegans* are viable and fertile, but show specific axon guidance defects of midline interneurons and commissural motoneurons[40]. Axon guidance defects can also be observed in worms lacking 6-O and 2-O sulfotransferase activity[40,41]. A careful analysis of the various neural phenotypes revealed that the individual HS-modifying enzymes are required for distinct, but partially overlapping, axon guidance and cell migration events[40]. These findings did not only provide the first evidence for a role of HS-modifying enzymes in nervous system development, but also implied that each modification is indeed required to convey specific information to developing neurons. This prompted Hobert and colleagues to postulate the existence of a "sugar code" specified by individual motifs present in a given HS, which could be regulated in a tissue-specific manner depending on the expression profile of HS-modifying enzymes and distinct HS core proteins. That glycosylation motifs can contain information was nicely demonstrated previously in the elucidation of the "glycan code" of the endoplasmatic reticulum[42]. There, Asn-linked glycans serve as maturation and quality control tags for proteins in the early secretory pathway and the carbohydrate composition encodes crucial indications about structure, localization, and age of synthesized glycoproteins.

The understanding of sugar biology has been lagging for many years because of major technical challenges. The good news is that many of these have been overcome in the last years. Certainly, the analysis of sugar function (glycomics) promises to shed light on numerous molecular mechanisms relying on glycosylated molecules. Nevertheless, in the case of HSPGs, there is accumulating evidence that their function depends not only on the HS sugar chains, but also on features of the corresponding core protein.

## HS CORE PROTEINS: SYNDECAN TUNES CELL MIGRATION SIGNALING

Syndecans are transmembrane proteins, whereas glypicans are attached to the cell surface via a GPI anchor. The cytoplasmic domain of syndecan is highly conserved and contains a PDZ-interaction motif at its C-terminus (Fig. 1). By contrast, the extracellular domain of syndecan shows only little sequence conservation. Mammalian syndecans can be cleaved off the cell surface by metalloproteinases, at least in cell culture experiments[2]. A physiological role for shed syndecan was demonstrated in mice, where soluble syndecan-1 regulates the chemotactic response of neutrophils in the inflammatory response[43]. Mammals have four syndecan and six glypican genes. In mice, the inactivation of a single syndecan gene does not result in obvious phenotypes. This is probably due to redundancy between the different family members, which can compensate partly for each other's function[2,44]**.** In contrast, the *C. elegans* and *Drosophila* genomes encode a single syndecan and contain only one (*C. elegans*) or two (*Drosophila*) glypican genes. Their simplicity has been exploited in the last 2 years to study the function of different HS core proteins in neural development[13,45,46,47,48].

The analysis of a *C. elegans* syndecan *sdn-1* null mutant revealed a role for this HSPG in neuronal migration[47]. In the worm, SDN-1 is highly expressed in neurons during early development and is also detected at lower levels in the nervous system of adult animals[47,49]. In the absence of SDN-1, several groups of neurons fail to migrate or stop prematurely on their migration route. Interestingly, similar neuronal migration defects are found in *sdn-1* mutants that express a truncated syndecan lacking the two major HS attachment sites[47,49]. In these mutants, SDN-1 is not detectable anymore by an anti-HS antibody, but staining with an antibody directed against a conserved phosphorylation site in the SDN-1 intracellular domain shows that the expression pattern of at least the phosphorylated form is not affected by the truncation[49]. These findings strongly suggest that it is the lack of HS, rather than loss of the syndecan core protein, that is responsible for the observed migration defects of neurons. Expression of a *sdn-1* cDNA under the control of a pan-neuronal promoter is sufficient to restore correct migration in *sdn-1* mutants, suggesting a cell-autonomous function of SDN-1 in neuronal migration[47].

Several groups revealed that neuronal migration requires specific modifications of the HS chains. For example, lack of 2-O sulfation motifs on HS significantly impairs neuronal migration in *C. elegans*[40,41,47]. Since *C. elegans* offers the possibility to build double or triple mutants of HS core proteins and different HS modifying enzymes, it is possible to address more challenging questions, such as which combination of modifications are needed on a given HSPG member (and at what time) to regulate a specific guidance choice of a neuron *in vivo*. First studies of this type indicated that at least one additional HS core protein, which carries 2-O sulfated and epimerized HS, teams up with syndecan to ensure the correct migration of neurons[47]. The identity of this additional HSPG remains to be determined.

Remarkably, syndecans might also be involved in the invasive behavior of transformed cells since these PGs are often deregulated in tumors[50,51]. Indeed changes in syndecan levels influence the adhesion between cells in culture[52,53]. The genetically amenable model organisms will hopefully permit a more detailed analysis of the biological underpinnings of HS-dependent cell migration.

## CELL SURFACE HSPGs FUNCTION IN AXON GUIDANCE AND SYNAPSE MORPHOGENESIS

A role for HSPGs in nervous system development was first reported in 1992, when Wang and Denburg showed that adding heparinase, an enzyme that degrades HS, to the culture medium of developing cockroach embryos caused guidance errors of several pioneer axons. They obtained the same effect when adding exogenous HS chains as competing molecules for putative HS ligands, whereas addition of other GAGs did not disturb axonal pathfinding[54]. Similarly, optic nerve fibers in *Xenopus* failed to reach their target area in the optic tectum when exposed to HS showing a high affinity for FGF-2, whereas HS with a high affinity for FGF-1 had no effect[55].

More recent efforts have concentrated on determining which family of HSPGs are implicated in axon guidance and responsible for the observed effects. From these studies, the HS core protein syndecan clearly emerged as a key player in nervous system development[13,17,45,46,47,48]. Syndecan null mutants of *Drosophila* and *C. elegans* both show midline axon guidance defects[13,45,47]. In addition, loss of syndecan affects embryonic development of motoneurons in worms as well as in flies[17,47] and axon guidance in the fly visual system[46]. Similar to the findings on neuronal migration, syndecan was found to act cell-autonomously in neurons to mediate axon guidance at the *Drosophila* midline[13,45], but showed a cell-nonautonomous way of interaction with LAR to control the patterning of *Drosophila* embryonic motoneurons[17]. Genetic removal of glypican in flies and worms does not cause the characteristic midline guidance phenotypes seen in syndecan mutants[45; Rhiner, C. and Hengartner, M., unpublished results], but the *Drosophila* glypican gene *dally-like* (*dlp*) is required for axon guidance in the fly visual system[46].

What about extracellular HSPGs in axon guidance? There is some evidence that agrin might also participate in axonal pathfinding: an agrin gradient can provoke repulsive growth cone turning of cultured *Xenopus* spinal neurons[56]. Although agrin-deficient mice show defects in terminal arborization of nerves[57], there is no direct evidence for a function of agrin during axon guidance *in vivo*. Finally, examined perlecan mutants in flies and worms do not display midline guidance errors[45; Rhiner, C. and Hengartner, M., unpublished results]. This observation has to be taken with caution, however. To evaluate the effect of loss of perlecan UNC-52 function in *C. elegans*, only *unc-52* RNAi and mutations that affect a subset of the alternatively spliced perlecan/UNC-52 forms could be analyzed, since strong *unc-52* alleles cause early embryonic lethality. In summary, cell surface–bound HSPGs, especially syndecan, contribute critically to correct axon guidance in genetic model organisms. It will be interesting to see whether syndecans and glypicans also regulate axon guidance in higher vertebrates.

Recent findings in the fly have revealed a novel role of syndecan and Dlp for different aspects of synapse organization[48]. Both cell surface HSPGs colocalize at *Drosophila* neuromuscular junctions (NMJs) and appear to control distinct synaptic processes; syndecan promotes the growth of presynaptic terminals, whereas Dlp is implicated in active zone formation required for normal electrophysiologic responses. Because the RPTP LAR regulates NMJ growth and active zone structure in *Drosophila*[58], and was shown to directly bind to syndecan and glypican[17,48], double mutant analysis was performed to elucidate the underlying signaling pathway. Epistasis experiments, combined with assaying LAR signaling output by phosphorylation of Enabled, suggested a model in which syndecan and Dlp compete for LAR binding to promote or inhibit its activity, respectively[48]. These findings represent the first evidence that distinct HSPGs might function as competitive ligands to modulate signaling in the synapse. It was already known for a long time that the HSPG agrin is crucial to induce the postsynaptic clustering of acetylcholine receptors in synaptogenesis[57], but it seems now that cell surface HSPGs also play an important part in synapse formation.

## REGULATION OF SLIT/ROBO SIGNALING BY SYNDECANS

How does syndecan contribute to axon guidance? Genetic interaction analyses in *Drosophila* and *C. elegans* have suggested that syndecan acts in the Slit-Robo signaling pathway. Consistent with this hypothesis, Slit can bind to HS *in vitro*[11,12,13]. In *Drosophila*, the introduction of one copy of a mutant allele for a robo receptor (robo or robo2) or slit into a syndecan loss-of-function background causes defects similar to those observed in the homozygous robo mutants[13,45]. Moreover, complete loss of Slit function in syndecan null worms does not further increase midline-crossing errors compared to either single null mutant, suggesting that syndecan and Slit act in the same signaling pathway[47].

Slits are conserved repellent guidance cues, secreted by cells at the midline to prevent axons expressing the Slit-receptor Robo from crossing the midline. But what is the role of syndecan in this signaling event? Based on the above findings, HS on syndecan has been proposed to act as molecular antennae on neurons, helping to modulate the reception of the Slit signal that is broadcasted by the midline cells. The HS sugar antennae could, for example, influence the efficacy of the signaling event (quantitative modulation) by binding to and locally increasing the concentration of the diffusing Slit signal. Syndecan mutant flies indeed show a different extracellular distribution of extracellular Slit compared to the wild type, which could be the cause of impaired signal transduction by Robo receptors and consequent midline guidance errors[13]. On the other hand, overexpression of *Drosophila* syndecan in neurons or in midline cells does not disturb Slit signaling[45]. This observation suggests that HS chains must do more than just serve as ligand gatherers for a limiting guidance cue. Another possibility is that HS is required to modulate the specificity of the Slit/Robo interaction (qualitative modulation). Indeed, certain HS modifications are crucial for some guidance choices but not for others[40,47]. It is therefore possible that a ligand needs to be presented to the receptor in the context of a specific HS motif to achieve maximal signaling impact. It is tempting to speculate that a neuron could fine tune its response to a general signal by subtly changing the large number of distinct modification patterns that can be put onto HS. The "sugar code hypothesis" goes even further and suggests that specific HS modifications could provide instructional cues to "recruit" ligands and mediate their binding to the cognate receptor. Such a model predicts that the ectopic introduction of specific HS modifications in a given cell could lead to activation of a signaling pathway that is usually inactive in this cell.

To think that the function of HSPGs is solely mediated by the HS chains seems too simplistic however. Several studies provide evidence for core protein–specific functions. Although pan-neuronal overexpression of *Drosophila* glypican Dlp can compensate for loss of syndecan in certain axon guidance events[13,46], it increases defects in the context of synaptic development[48]. On the other hand, syndecan overexpression never seems to improve neural defects of *dlp* mutants[46]. It is therefore likely that many signaling events also depend on the nature of the HS core protein. In order to solve this question, future experiments will require comprehensive structure/function analysis of the various HSPGs to determine the regions that are crucial for regulation of guidance signaling.

Syndecans are extremely versatile HSPGs since their cytoplasmic domains also allow interaction with intracellular binding partners. Several PDZ-domain proteins, such as syntenin, CASK/LIN-2, synbindin, and synectin[59,60,61,62,63], have been shown to bind to the C-terminus of syndecans, although the *in vivo* relevance of these interactions still needs to be further characterized. Phosphorylation of the syndecan cytoplasmic domain was found to be essential for inside-out signaling that specifies the left-right looping of the heart and gut in *Xenopus*[64]. Although a phosphorylated form of syndecan SDN-1 has been found to be prominently expressed at the nerve ring of *C. elegans*[49], direct evidence for syndecan-dependent inside-out signaling in nervous system development is still lacking.

Neurons seem to require syndecan to modulate their response to guidance cues present in the extracellular matrix. This regulation needs to be dynamic and is most likely achieved through a combinatorial control of HS-modifying enzyme expression, syndecan cell surface exposure, and shedding by metalloproteinases. Different syndecan family members, as well as the HS-modifying enzymes, indeed show tightly regulated spatiotemporal expression patterns[65,66] underscoring their suitability as dynamic regulators of axon guidance signaling.

Despite the emerging evidence that differentially modified HS could endow individual neurons with unique HS fingerprints to regulate their sensitivity to ligands, they are not the only molecules capable of generating a vast number of structurally different forms. Neurexins, cadherins, and cadherin-related neuronal receptors also show a high degree of molecular diversity, generated via extensive alternative splicing[67,68,69]. An even more striking example is the *Drosophila* gene Dscam, which can potentially generate 38,016 mRNA forms through alternative splicing[70]. This large diversity of isoforms is required for the correct wiring of neurons in the somatosensory system of the fly[71]. According to the chemoaffinity hypothesis, proposed by Roger Sperry in 1963, the high degree of specificity by which neurons connect to each other must either derive from a enormous number of different affinity labels or from a small set of signals that provide positional information by forming overlapping concentration gradients[72]. The discovery of HSPGs as regulators of axon guidance signaling indicates that reality might lie somewhere between these two extremes, and that specificity can be achieved by combining gradients of relatively few guidance cues with highly variable "coreceptors" that fine tune the responses of cells to these gradients.

## CONCLUDING REMARKS

Dedicated biochemical and genetic efforts have greatly enhanced our knowledge about the complex structure of HS and its diverse roles in many developmental processes including neuronal migration, axon guidance, and synapse formation. Nevertheless, there is still much that we do not know. For example, distinct molecular mechanism have been proposed for the role of HSPGs in different neuronal contexts including functions as receptors, coreceptors, (competitive) ligands, and as molecules that function to transport or enrich ligands on the cell surface or that target receptors to specific cell membrane domains such as lipid rafts[73]. What determines which mechanism a given HSPG will utilize? What are the specific motifs present in the molecular glyco-antennae of HSPGs? The ligand-binding sequence on HS is only known for very few factors. More sophisticated analysis of HS composition and ligand-binding sites will be a key to understand the regulatory role of HSPGs, such as syndecan, and set the basis for the design of inhibitors. What are the functions mediated by the core proteins? Who do they talk to? Elaborate genetic screens will certainly allow us to isolate further components of HSPG-dependent signaling. Finally, from an evolutionary and medical point of view, it will be interesting to learn whether these versatile glycoproteins also contribute to nervous system development and function in higher vertebrates. Much exciting research remains to be done.

A very recent study provides evidence that syndecans are not only important for neuronal migration in *C. elegans* but also participate in several neuronal migration events in mammals, underlining a conserved function: careful reexamination of the brain phenotype in syndecan-3 knock-out mice revealed impaired radial migration in the cortex as well as neuronal migration defects in the rostral migratory stream [74].

## ACKNOWLEDGMENTS

This work was funded by the Swiss National Science Foundation (MOH), the Ernst Hadorn Foundation (MOH), and the Forschungskredit of the University of Zurich (CR).
